# Oxidative Stress Modifies the Levels and Phosphorylation State of Tau Protein in Human Fibroblasts

**DOI:** 10.3389/fnins.2017.00495

**Published:** 2017-09-07

**Authors:** Alejandro Ibáñez-Salazar, Bernardo Bañuelos-Hernández, Ildefonso Rodríguez-Leyva, Erika Chi-Ahumada, Elizabeth Monreal-Escalante, María E. Jiménez-Capdeville, Sergio Rosales-Mendoza

**Affiliations:** ^1^Laboratorio de Biofarmacéuticos Recombinantes, Facultad de Ciencias Químicas, Universidad Autónoma de San Luis Potosí San Luis Potosí, Mexico; ^2^Sección de Biotecnología, Centro de Investigación en Ciencias de la Salud y Biomedicina, Universidad Autónoma de San Luis Potosí San Luis Potosí, Mexico; ^3^Facultad de Medicina, Universidad Autónoma de San Luis Potosí San Luis Potosí, Mexico

**Keywords:** Alzheimer's disease, fibroblasts, tau protein, oxidative stress, phosphorylation state

## Abstract

Since the tau protein is closely involved in the physiopathology of Alzheimer's disease (AD), studying its behavior in cellular models might lead to new insights on understanding this devastating disease at molecular levels. In the present study, primary cultures of human fibroblasts were established and used to determine the expression and localization of the tau protein in distinct phosphorylation states in both untransfected and tau gene-transfected cells subjected to oxidative stress. Higher immunopositivity to phospho-tau was observed in cell nuclei in response to oxidative stress, while the levels of total tau in the cytosol remained unchanged. These findings were observed in both untransfected cells and those transfected with the tau gene. The present work represents a useful model for studying the physiopathology of AD at the cellular level in terms of tau protein implications.

## Introduction

Neurodegenerative disease is a general term for a range of conditions that primarily affect neurons in the brain, which is characterized by degeneration and/or progressive death of nerve cells leading to dementia and/or movement impairments (Seung-Jae et al., 2011). Dementia seriously affects the ability of performing daily activities and is a common sign of various diseases, including Alzheimer's disease (AD); which accounts for approximately 60–70% of dementia cases and constitutes the most common neurodegenerative disease in the elderly (Qiu et al., 2009; Xiao-Hong et al., 2012). One of the common characteristics of neurodegenerative diseases is the progressive accumulation of aggregates of specific proteins in the brain with a particular regional pattern for each disorder. As the diseases progress protein aggregates spread among brain cells, eliciting both microglial inflammatory responses and neuronal death (Muramatsu et al., 2008). AD is characterized by the extracellular deposition of aggregates of β-amyloid (Aβ) (Yoshida and Ihara, 1993) forming senile plaques, in addition to the intraneuronal accumulation of hyperphosphorylated tau protein as neurofibrillary tangles (Selkoe, 2004; Braak et al., 2006; Hardy, 2006). Several neurodegenerative diseases share with AD tau misfolding and are recognized as taupathies. Recent research has focused on studying the factors leading to tau aggregation and apparent spreading in taupathies (Spillantini and Goedert, 2013).

Tau is a cytoplasmic protein, described in neurons and other cell types (Cross et al., 2000). The first cell function described for tau was its role in the assembly of microtubule cytoskeleton (Wang et al., 1997). In HeLa cells and fibroblasts, tau is localized in the nucleolus; where it is associated with regulatory regions of this organelle. It has been suggested that tau plays a role in the nucleolar organization and heterocromatization (Sjoberg et al., 2006). With regard to the chromatin binding function, *in vitro* studies have shown that purified tau binds to AT rich DNA regions with higher affinity than GC rich regions (Hua and He, 2003). Another report has shown an association of chromosomal aberrations with a mutation of tau existing in cultured fibroblasts and lymphocytes from patients with AD (Rossi et al., 2008).

Audrey et al. (2011) observed in neuron culture assays, subjected to oxidative stress, an accumulation of dephosphorylated tau in the nuclei. Using immunoprecipitation assays, the ability of tau to interact with neuronal DNA under thermal stress was proven. In addition, in cells overexpressing tau, comet assays revealed that tau exerted DNA protective effects against free radical-induced damage. These findings have relevant implications on understanding the pathology of AD since oxidative stress and DNA damage play a key role in this disease (Kruman et al., 2004). In addition the localization of tau in the nucleus, specifically in the nucleolus, suggested that tau aggregates may have a role on gene expression mediated by epigenetic mechanisms (Frost et al., 2014).

Frost et al. (2014) reported changes in the chromatin structure associated to tau expression, suggesting the role of tau in the epigenetic changes occurring in neurodegenerative diseases. A model of transgenic Drosophila expressing a tau mutation associated to dementia and parkinsonism (FTDP-17 human gene) was used to demonstrate that tau promotes a shift from heterochromatin to euchromatin resulting in aberrant gene expression that contributes toward neurodegeneration. In addition, oxidative stress was involved not only in DNA damage but in heterochromatin loss, which suggested aberrant gene expression as the link between oxidative stress and neurodegeneration.

Several models to study the behavior of tau at cellular levels have been described, including mouse skin fibroblasts and recombinant cell lines (Fraser et al., 2009). In humans, fibroblast lines have also been used to study Aβ and tau aggregation (Auburger et al., 2012). With the purpose to open new paths in understanding the physiopathology of AD, the present study aimed at evaluating the behavior of both native and overexpressed tau under oxidative stress in human fibroblasts.

## Materials and methods

### Fibroblast isolation and culture

Fibroblast cultures were established from skin biopsies (neck region behind the ear) of healthy subjects. This procedure, approved by the ethics committee from the “Ignacio Morones Prieto” Central Hospital (protocol number 32-13), was performed by qualified staff from the Department of Neurology of the same hospital. All study subjects granted the written informed consent.

To establish fibroblast primary cultures, a biopsy from a healthy subject was collected and transported in collection medium for tissue biopsies (50 mL of growth medium containing: 125,000 U/L Penicillin, 125 mg/L streptomycin, and 50 mg/L kanamycin; Freshney, 2010). Once under the tissue culture hood, the biopsy was washed twice in a 50 mL conical sterile tube using 20 mL of PBS supplemented with antibiotic/antimycotic mix (100 U/mL penicillin, 100 μg/mL streptomycin, and 5 mg/mL amphotericin; SIGMA, San Luis, MO, USA). Tissues were subsequently cut off into 5–10 mm pieces with a scalpel in the presence of 0.5 mL of PBS. Tissue sections were placed in 12-well plates (3–4 fragments per well) containing 2 mL of DMEM-F12 (SIGMA, San Luis, MO, USA) supplemented with 10% calf serum and antibiotic/antimycotic mix. Cultures were maintained at 37°C, 5% CO_2_ and 95% humidity. Cells were screened for contamination-culture progress by microscopy with subcultures every 3–4 days. Fibroblasts were harvested by incubation with 0.25% trypsin (SIGMA, San Luis, MO, USA) at 37°C for 2–10 min. Then, medium with 10% fetal bovine serum was added and the cell suspension was transferred to a 15 mL conical tube. The suspension was centrifuged at 2,000 rpm for 5 min and, after washing with fresh medium, transferred to a new plate. For cryopreservation 1 mL of cell suspension, containing 1.0 × 10^6^ cells in culture medium supplemented with 10% CS and antibiotics, was mixed with glycerol to reach 10% concentration and frozen at −80°C. Cell stocks were used to set fibroblast cultures used for evaluating cell viability and the behavior of Tau under overexpression and oxidative stress exposure, in two independent experiments run in triplicate.

### Tau localization by immunohistochemistry

Cells were grown in flasks with culture medium (DMEM-F12 + 10% CS + ST) and after reaching 80–90% confluence they were trypsinized (harvested), washed with 1 × PBS, and seeded in 24-well plates containing a circular glass 13 mm-coverslip that facilitated transferring and staining of the cells. Coverslips were pretreated with a 20 mg/mL poly-L-lysine solution for 12 h at 37°C and subsequently washed once with 1 × PBS, twice with sterile distilled water, and finally exposed to UV light for 20 min. Once cells were seeded onto coverslips, they were cultivated for 2 days. Cells were fixed with 300 μL of cold 4% paraformaldehyde for 20 min and washed 3 times with PBS for 10 min. Cells were incubated for 2 h in blocking solution (1 × PBS plus 10% Horse serum and 0.25% Triton X-100). Primary labeling was performed overnight at 4°C under stirring and the secondary antibody was subsequently added at a 1:10,000 dilution for 2 h at room temperature; the coverslips were protected from light during the rest of the technique. Coverslips were incubated in Hoechst 33258 (Bis-benzamide) solution (2 mg/mL) for 15 min and, after washing, they were mounted with Vectashield (2.5%) onto slides upside down. After drying, the preparations were sealed with enamel and stored at 4°C. Slides were analyzed using an Axio Lab A1 (Zeiss, Oberkochen, Germany) fluorescence microscopy (Ex 405 nm/Em 488 nm for GFP and Ex 358 nm/Em 461 nm for DAPI). Mean fluorescence intensity (MFI) was quantified in 9 microphotographs (15 well-defined cells per photograph) of each condition using the Zen Lite 2012 software (Karpenko et al., 2015; Macedo et al., 2017). MFI data analysis was performed by ANOVA followed by Tukey test using the GraphPad Prism 5 software (GraphPad Software Inc., San Diego, CA, USA).

Tau protein was evidenced in cultured fibroblasts using commercial monoclonal antibodies and also through an anti-human phosphorylated tau anti-serum produced in mice. In order to label total tau protein, mouse monoclonal anti-tau (Tau5; MA5-12808, ThermoFisher Scientific, Rockford, IL, USA) was employed and to detect phosphorylated tau protein a rabbit monoclonal anti-tau (pSer396; PHF, Abcam, Cambridge, MA, USA) and a mouse monoclonal antibody (pSer202+pThr205; AT8, MN1020, Thermo Scientific, Rockford, IL, USA) were used. AT8 epitope was also labeled using an *ad hoc* anti-serum produced in mice as described below.

### Development of anti-tau in mice

Animals were maintained under standard laboratory conditions with free access to food and water following procedures indicated by the Federal Regulation for Animal Experimentation and Care (SAGARPA, NOM-062-ZOO, 1999, Mexico); approved by the Institutional Animal Care and Use Committee. Thirteen week-old female BALB/c mice were used. A phospho-202, 205 tau synthetic peptide corresponding to tau 199–207 (SPG{PSER}PG{PTHR}PG) was used to generate hyperimmune serum (Invitrogen, Carlsbad, CA, USA). Ten micro grams doses of the tau peptide were emulsified in either complete Freud's adjuvant (CFA) or incomplete (IFA).

Two mice were immunized on day 1 into the rear footpad with 10 μg of tau peptide emulsified in 20 μL of CFA. Three subsequent doses were intraperitoneally administered on days 8, 15, and 22; consisting of 50 μg of tau peptide emulsified in one volume of IFA. Mice were bled on day 29 to measure antibody titers. Animals were subsequently sacrificed by spinal dislocation and blood was collected by cardiac puncture to measure seric anti-AT8 antibody titers by ELISA.

For ELISA, 96-well polystyrene plates were coated with tau peptide (1 μg per well). Plates were washed with PBS-T between each step of the protocol to remove residues from previous incubation. Plates were blocked with 5% fat-free milk and the serial dilutions of the test sera were applied (1:100 to 1:800). A secondary horseradish-conjugated anti-mouse IgG antibody was added (1:10,000 dilution). Finally, an ABTS substrate solution was added and OD values were measured using an ELISA microplate reader (Bio-Rad, Hercules, CA, USA). In order to evaluate whether the obtained serum is useful for the detection of phosphorylated tau in neurofibrillary tangles; immunohistochemical analyses were performed in autopsied brain tissue from AD patients and healthy subjects using standardized protocols (Rodríguez-Leyva et al., 2015).

### Oxidative stress assays

Confluent fibroblast cultures were subjected to oxidative stress by adding serum-free medium containing H_2_O_2_in the 20–1,000 μM range. One hour after incubation; cell viability was measured by the resazurin assay (O'Brien et al., 2000).

### Overexpression of tau protein

A synthetic version of the tau gene (441 aa isoform, GenBank Acc. No. NM_005910.5) containing the flanking *EcoR*I and *Apa*I restriction sites was obtained from GenScript (Piscataway, NJ, USA). The tau gene was provided into the pUC57 cloning vector and was subcloned into the pVAX1™ (Invitrogen, Carlsbad, CA, USA) by conventional restriction ligation techniques to generate the vector pVAX-Tau. A positive clone confirmed by restriction profile and sequencing was propagated in *E. coli* to isolate the plasmid for transfection assays using the QIAprep Spin Miniprep Kit (Qiagen, Hiden, Germany).

1 × 10^5^ fibroblasts were seeded in 24-well plates and grown until reaching 90% confluence. For transfection the Escort III reagent was employed at a concentration of 1 μg/mL; diluted in 100 μL of DMEM F12 culture medium without serum or antibiotics and mixed with 100 μL of pVAX-Tau DNA solution to reach a final plasmid concentration of 300 ng/μL. Escort III reagent/DNA mix was incubated at room temperature for 30 min. Medium was removed from the cultures and Escort-DNA complex mixture was added along with 800 μL of DMEM F12 medium, and incubation was run for 18 h. The transformation mixture was subsequently removed from cultures and fresh medium supplemented with antibiotics and serum was added, the resulting cultures were incubated for 24 h to verify transfection. pVAX1 carrying the β-galactosidase gene was used as a transfection control, thus the cells were subsequently incubated with X-gal developing solution to verify the transfection procedure.

### In gel anti-tau western blot

For western blot analysis, pellets containing ~2 × 10^6^ test cells were resuspended in 20 μl of Laemmli 1 × buffer and incubated at 95°C for 10 min. Protein extracts were resolved by SDS-PAGE using 4/12% polyacrylamide gels under denaturing conditions. The resulting gel was washed with a 50% isopropanol solution containing 5% acetic acid for 15 min and subsequently washed with HPLC water for 15 min. Gels were blocked by incubation in 5% BSA solution containing 0.1% Tween 20 for 2 h. Primary labeling was performed by overnight incubation at 4°C with either Tau5 or anti-β-actin antibodies at a 1:2,000 dilution prepared in 5% BSA plus 0.1% Tween 20. Gel was washed three times with 1 × PBS plus 0.1% Tween 20, with a 10 min incubation in between. Secondary labeling was done with a CF™ 488A-labeled secondary antibody (Biotium, Fremont, CA, USA) at a 1:2,000 dilution, in which the gel was incubated for 2 h at room temperature and protected from the light. The gel was subsequently washed 3 times with 1 × PBS plus 0.1% Tween 20 for 10 min and once with 1 × PBS for 10 min. The gel was scanned in a LI-COR's Odyssey CLx equipment (Lincoln, NE, USA).

## Results

### Primary fibroblast cultures express different tau forms in both nucleus and cytosol

Titration of the anti-AT8 hyperimmune serum, raised in mice, revealed titers of 1:320 in ELISA targeting the AT8 synthetic peptide. To validate the reactivity and specificity of the AT8 anti-serum, immunohistochemistry analyses were applied on autopsied brain tissue from AD patients. Strong reactivity was observed in such samples, at similar level to the commercial AT8 antibody; whereas biopsies from healthy subjects showed no significant reactivity (Figure S1).

**Figure 1:**
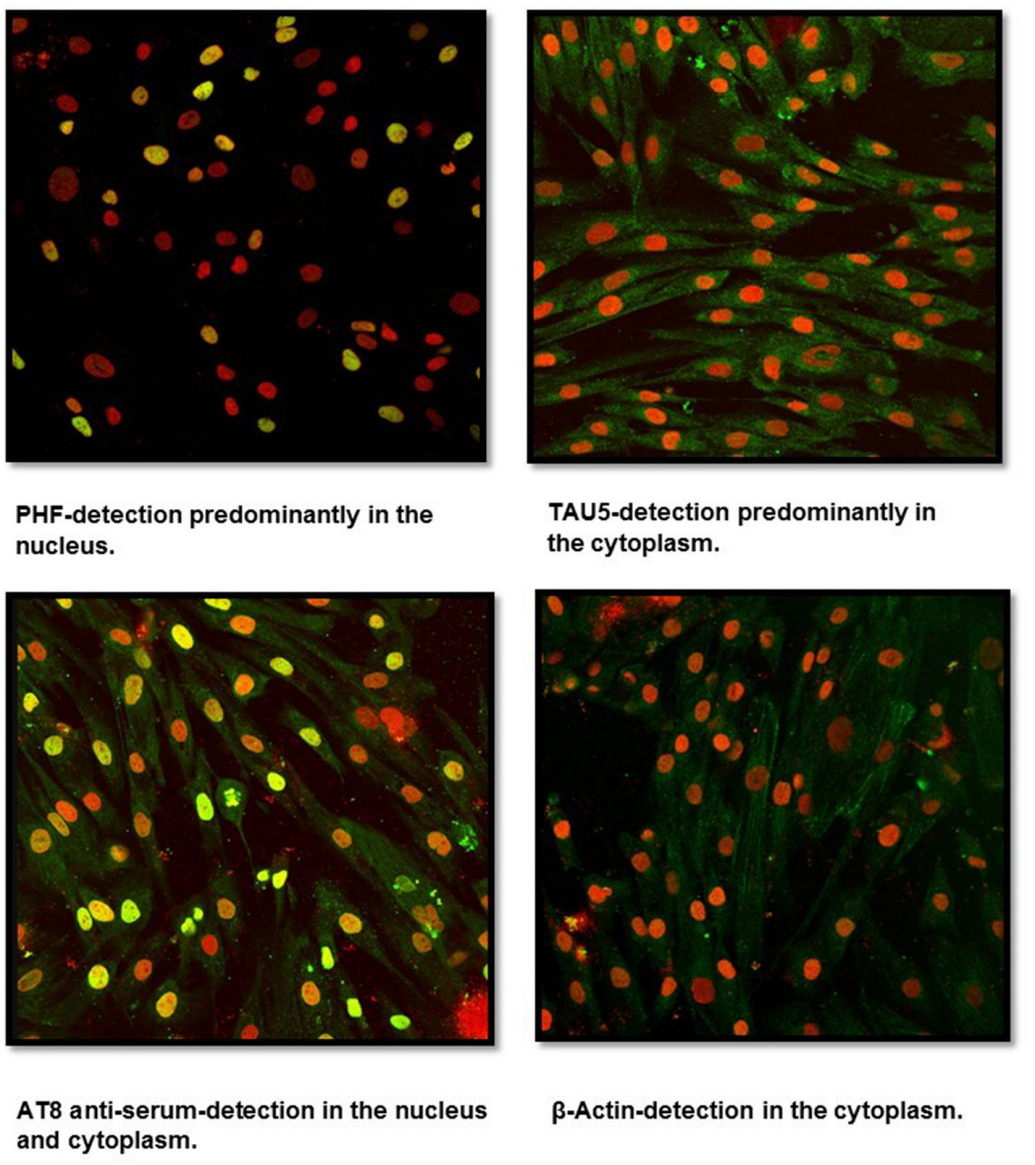
Confocal microscopy analysis of untransfected human fibroblasts. Cells isolated from a skin biopsy showed a positive reactivity for Tau5 (cytoplasm), PHF (nucleus), and AT8 anti-serum (cytoplasm and nucleus). Nuclei were stained with Sytox (in red), while Tau was detected through FITC conjugated to the secondary antibody (in green). Sytox/FITC signal overlap is observed in yellow.

Fibroblast cultures were successfully established from healthy subject skin biopsies using the DMEM F12 medium. Labeling with anti-tau antibodies (PHF, Tau5, AT8 serum, β-Actin) revealed, using confocal microscopy, a positive reactivity for PHF and Tau5 antibodies as well as for AT8 serum (Figure 1). PHF antibody showed a predominant reactivity at the nucleus whereas a very low signal was observed in the cytoplasm. Tau5, which identifies total protein tau, showed a predominant signal in the cytoplasm. In contrast, anti-AT8 serum reacted in both the cytosol and the nucleus. Control labeling using the β-actin antibody showed only cytosolic positivity.

### Oxidative stress and overexpression of tau

**Figure 2:**
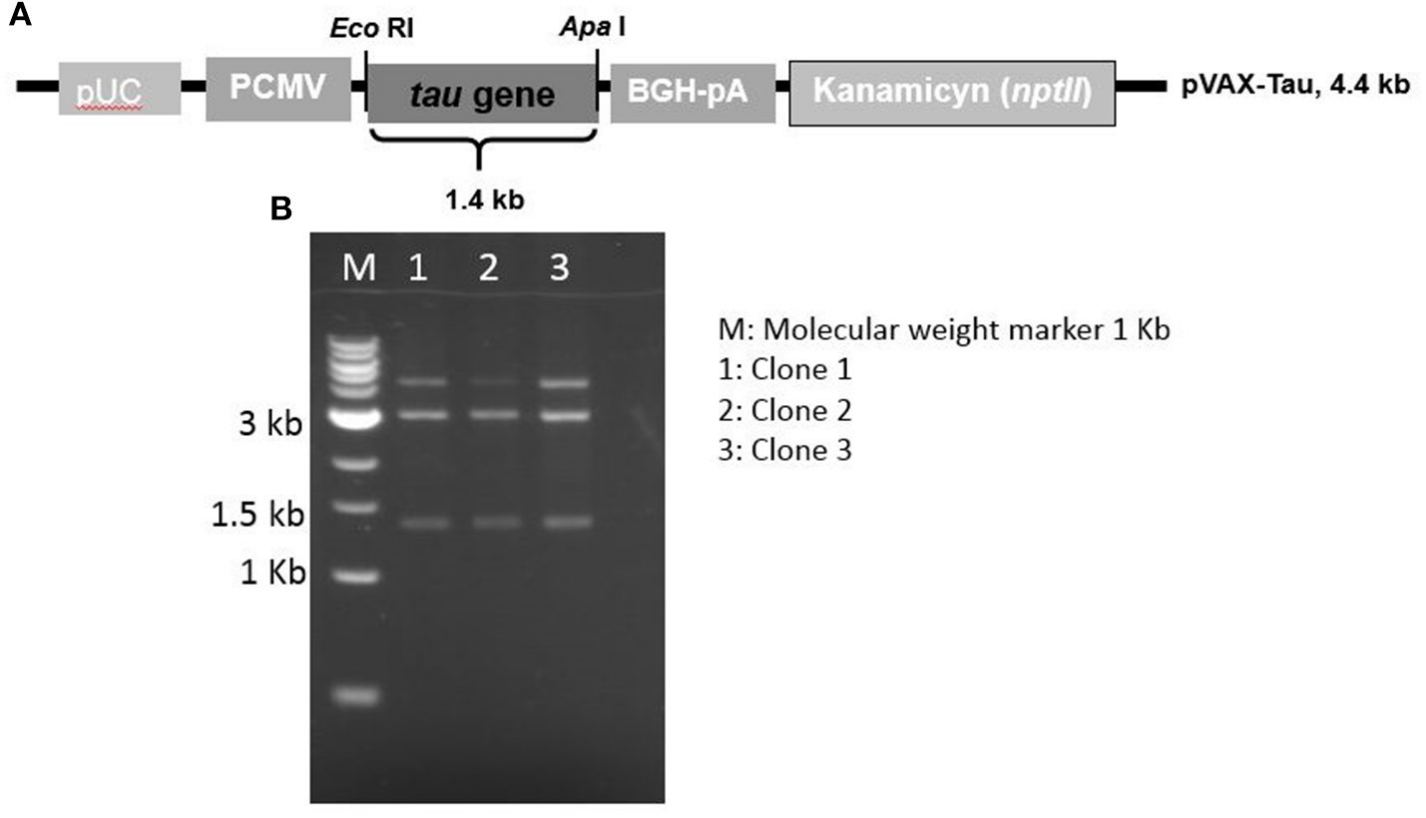
Map and restriction profile of the expression vector to overexpress the tau gene. **(A)** Physical map of the pVAX-Tau vector, which is based in the pVAX backbone and drives the expression of tau gene by the CMV promoter. **(B)** Restriction profiles of the constructed pVAX-Tau vector; the release of the 1.4 kb fragment upon EcoRI/ApaI restriction indicated the presence of the tau gene, which was confirmed by conventional sequencing.

A DNA vector, called pVAX-Tau, mediating tau overexpression was assembled using a synthetic gene under the control of the CMV promoter. A restriction profile confirming the pVAX-Tau vector is shown in Figure 2 along with the pVAX-Tau physical map. This vector was used to transfect fibroblasts. To study the behavior of tau under oxidative stress and overexpression, either untransfected fibroblasts (UnFs) or pVAX-Tau/transfected fibroblasts (TFs) were exposed to 20 and 60 μM H_2_O_2_. Fibroblast cultures with no H_2_O_2_ exposure were included as controls.

**Figure 3:**
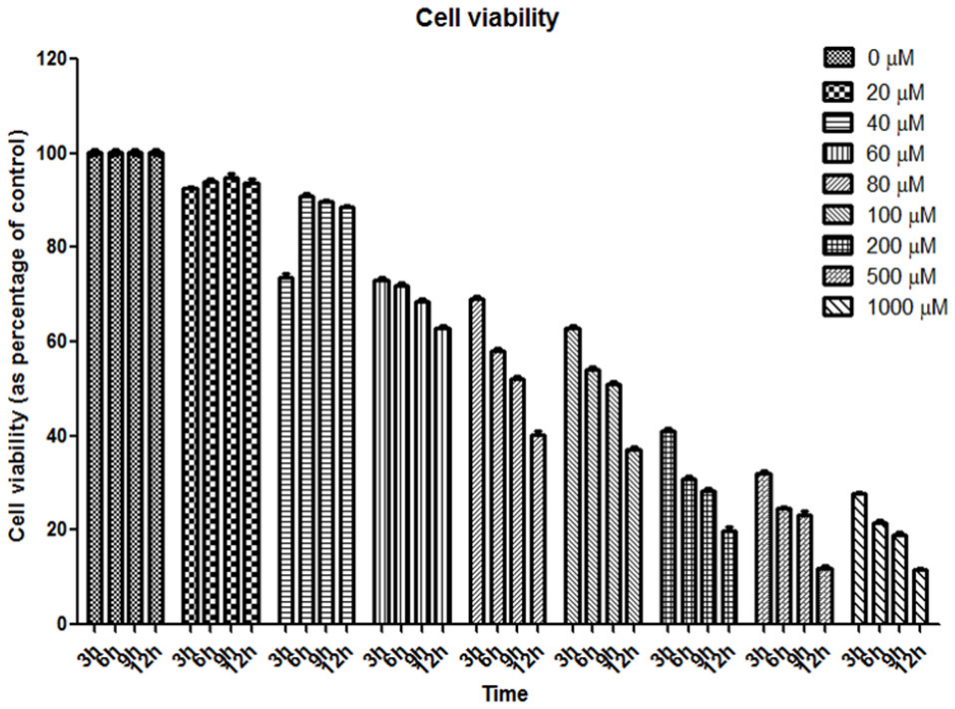
Cell viability assay of human fibroblasts exposed to oxidative stress. Graph of resazurin experiment. Eight concentrations of hydrogen peroxide at four-time points were assessed. Cell viability is reported as percentage of control (considered as 100%).

Fibroblasts were exposed to varying concentrations (20–1,000 μM) of H_2_O_2_ during 3, 6, 9, and 12 h (Figure 3). Cell viability assays revealed that exposure to 200, 500, and 1,000 μM H_2_O_2_ led to high cell death rate (37, 28, and 25% in viability; respectively); whereas exposure to 20 and 60 μM H_2_O_2_ led to a moderate stress since viability values under these conditions were 92 and 72%, respectively (Figure 3). Therefore, exposure to 20 and 60 μM H_2_O_2_ was selected to assess the behavior of tau.

To observe the effect of oxidative stress on the behavior of tau protein, three commercial antibodies were used (anti-PHF that identifies the phosphorylated protein, anti-Tau5 that identifies the total tau protein, and anti-AT8 that identifies a characteristic epitope of the phosphorylated-pathologic protein). In addition, the serum obtained from mice immunized with the AT8-Tau synthetic peptide was used to observe its behavior during oxidative stress and to compare with commercial antibodies.

**Figure 4:**
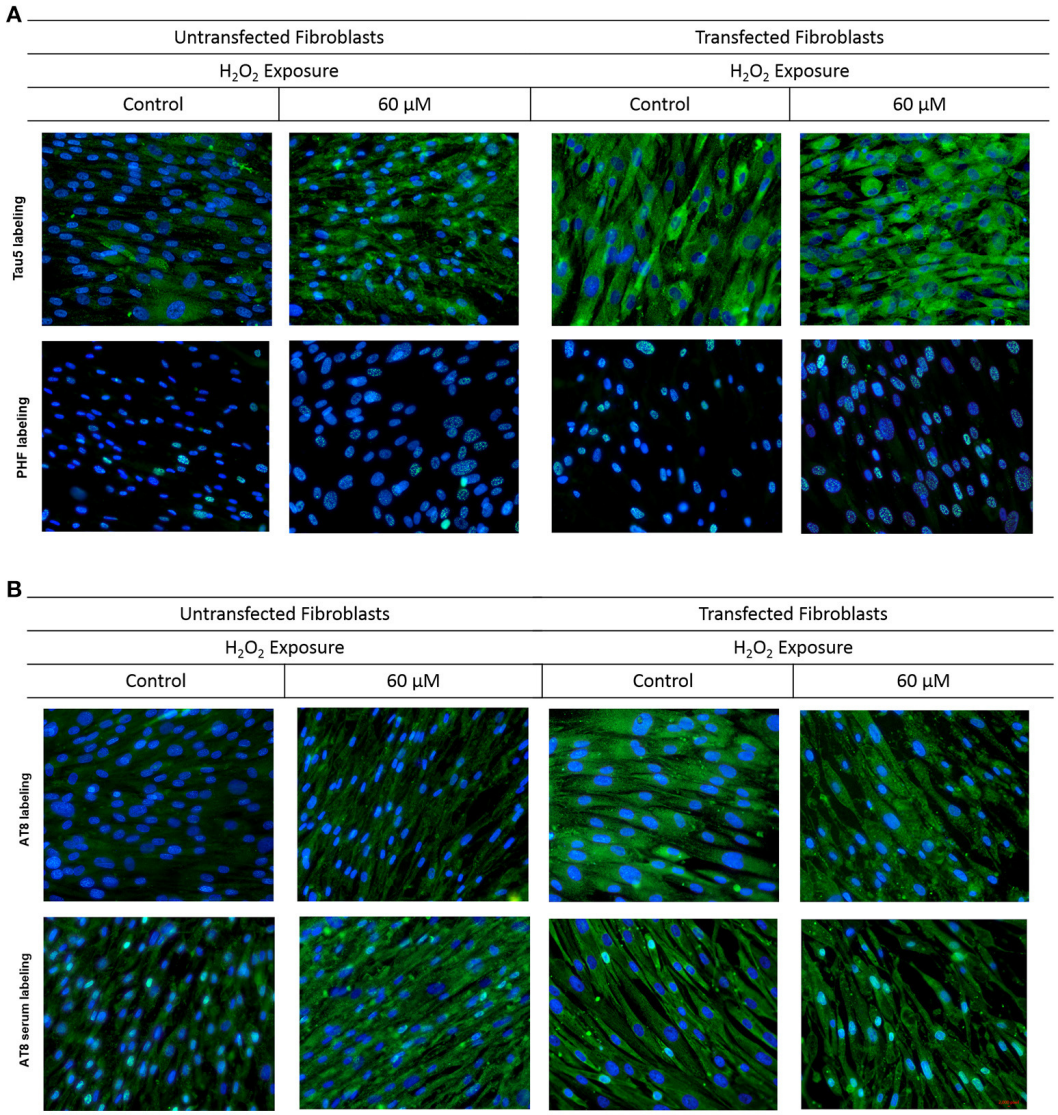
Behavior of tau in fibroblasts upon oxidative stress and overexpression. Fibroblasts cultures were subjected to transfection with the tau gene and exposed to H_2_O_2_ to compare the behavior of Tau to that of untrasfected fibroblasts (UnFs). **(A)** Tau5 labeling revealed increased cytoplasmic reactivity in TFs exposed to 20 and 60 μM H_2_O_2_. PHF reactivity in UnFs was located into the nucleus and is increased in TFs. **(B)** AT8 labeling of UnFs revealed high cytoplasmic reactivity, which increased in TFs and appears into the nucleus. Anti-AT8 serum showed a similar pattern of that of the AT8 antibody but with a higher signal. Scale bar 2,000 pixels, equivalent to 100 μm. Nuclei were stained with DAPI (in blue), while Tau was detected through FITC conjugated to the secondary antibody (in green). DAPI/FITC signal overlap is observed in green/white.

**Figure 5:**
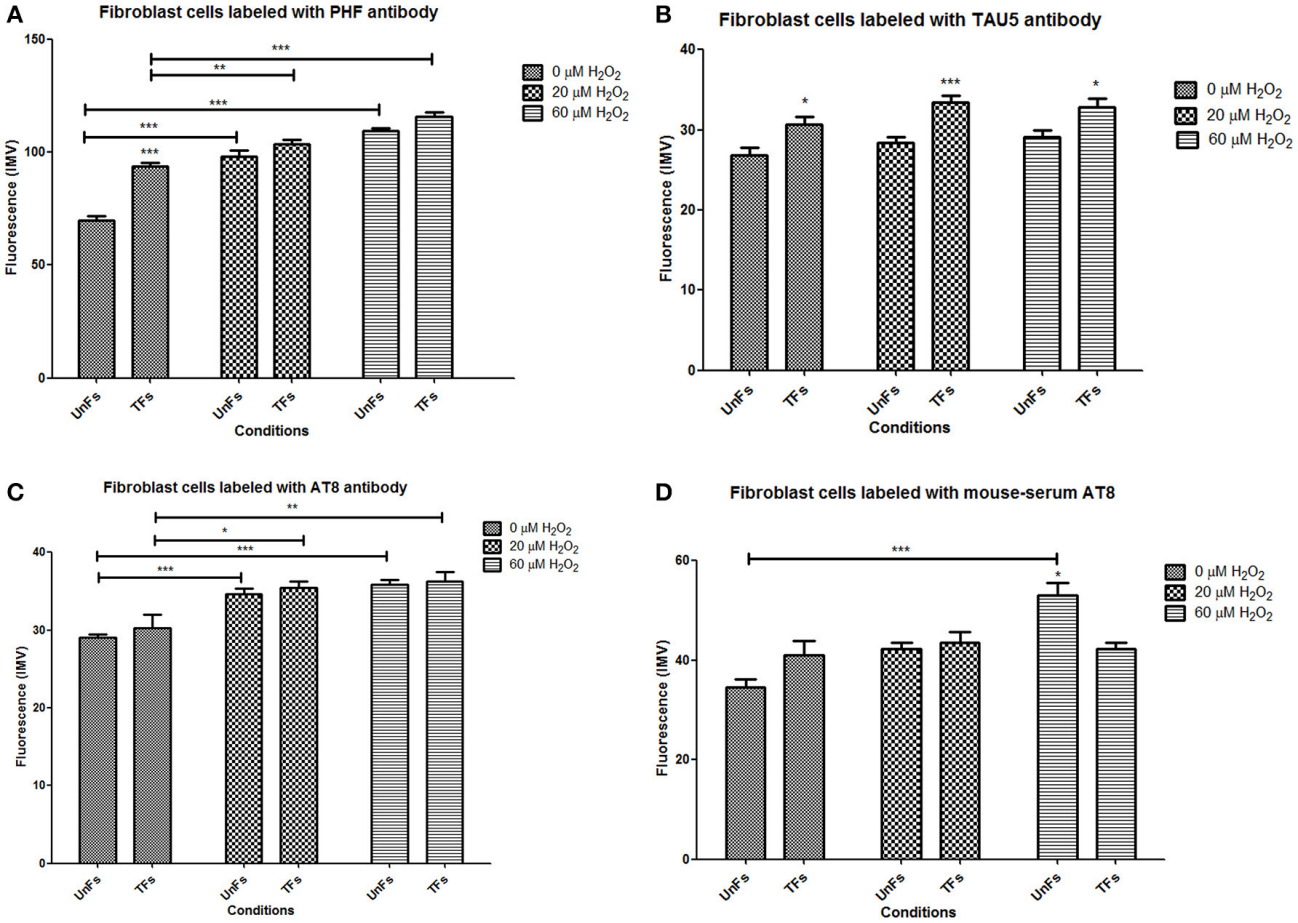
Different levels of distinct forms of Tau in fibroblasts. Quantification of mean fluorescence intensity in microphotographs corresponding to labeling with distinct anti-Tau antibodies was performed with the Zen Lite 2012 software. Bars represent mean ± *SD* of a total of 135 cells counted in 9 microphotographies from each slide. **(A)** Nuclear levels of Tau detected with PHF antibody. **(B)** Cytosolic levels of Tau detected with Tau5 antibody. **(C)** Cytosolic levels of Tau detected with AT8 antibody. **(D)** Cytosolic levels of Tau detected with anti-AT8 serum. Asterisks right above columns indicate statistical significant differences between UnFs and TFs. Bars denote statistical significant differences between cells subjected to distinct H_2_O_2_ treatments. ^*^p = 0.01; ^**^p = 0.001; ^***^p = 0.0001.

UnFs showed a positive signal for the PHF antibody in both cytosol and nucleus (Figure 4A), with predominance in the nucleus; which is in agreement with findings of the initial study performed with confocal microscopy analysis. TFs showed higher signal upon PHF labeling. Such signals increased upon oxidative stress in either UnFs or TFs, with a higher effect in the 60 μM H_2_O_2_ treatment. However, in TFs subjected to H_2_O_2_ exposure nuclear levels of PHF tau increased, especially in cells exposed to 60 μM H_2_O_2_. In agreement with these observation, quantification of fluorescence in terms of MFI confirmed a significant increase in the amount of Tau immunopositivity located in the nucleus of TFs exposed to 60 μM H_2_O_2_. Although the 20 μM H_2_O_2_ treatment resulted in no significant increase in MFI values, a tendency toward an increase in nuclear immunopositivity as compared to baseline was observed in this condition (Figure 5A).

Labeling with Tau5 (total tau; Figure 4A) revealed a higher signal in TFs when compared to UnFs. Tau5 immunopositivity increased upon oxidative stress in both UnFs and TFs. An increase in the nuclear signal was observed in TFs upon 60 μM H_2_O_2_ treatment. Quantification of fluorescence upon labeling with Tau5 antibody confirmed the increase of Tau in TFs when compared to UnFs, with no significant modifications upon oxidative stress exposure (Figure 5B).

Reactivity to commercial AT8 antibody (Figure 4B) was positive in both UnFs and TFs, with predominance in the cytoplasm. Both UnFs and TFs showed an increase in AT8 reactivity upon oxidative stress, especially in the 60 μM H_2_O_2_ treatment. Similar reactivity patterns to those of the commercial AT8 antibody were observed when cells were labeled with the anti-AT8 tau serum (Figure 4B); nonetheless higher signals were verified. These observations were confirmed by fluorescence quantification data since higher values were recorded in both UnFs and TFs upon H_2_O_2_ exposure (Figure 5C). Similar findings were found upon labeling with the anti-AT8 serum (Figure 5D).

**Figure 6:**
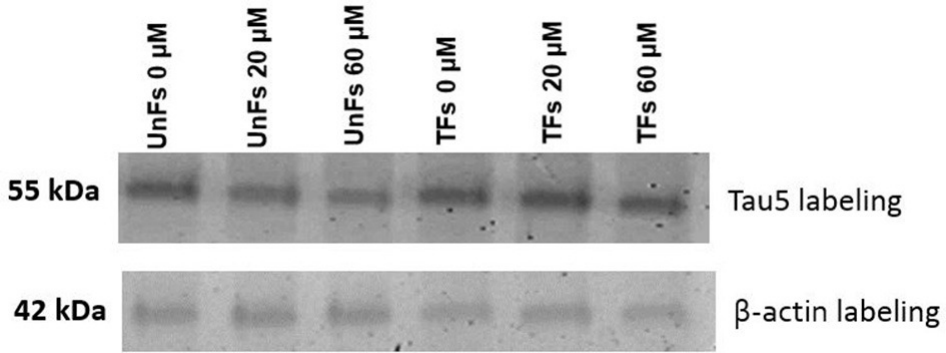
Assessment of Tau overexpression by Western blot. Total protein extracts from fibroblasts under distinct experimental conditions were subjected to immunodetection using Tau5 antibody. β-actin immunodetection was performed as loading control. UnFs, untransfected fibroblasts; TFs, fibroblasts transfected with pVAX-Tau.

A confirmatory analysis by Western blot was conducted to confirm the increase of total Tau levels in TFs. Total protein extracts from TFs showed increased levels of Tau upon labeling with the Tau5 antibody when compared to the signal of UnFs (Figure 6).

## Discussion

In the present study, primary fibroblasts cultures were used to study the expression of tau as a convenient model in the physiopathology of Alzheimer's disease. First an anti-AT8 serum was obtained using a phosphorylated peptide, showing strong reactivity in histological sections of patients having Alzheimer's disease (Rodríguez-Leyva et al., 2015) at a comparable level to that of the AT8 commercial antibody. In the future, this serum can be purified to serve as an alternative source of pathologic tau detection. This serum will be especially useful in experiments aiming at neutralizing prionic tau in fibroblast cultures that will require higher amounts of blocking tau antibodies in fibroblast cultures (Aguzzi et al., 2008; Woerman et al., 2016). Our group is currently working on determining if tau shows cell-to-cell propagation in fibroblast cultures using the anti-AT8 serum as a blocking approach.

Fibroblasts cultures were successfully established from a skin biopsy, collected from a 61-year old female subject; that allowed performing several analyses. The growth of our cultures showed similar behavior to that reported by other groups (Corlier et al., 2015). The behavior of tau upon oxidative stress was evaluated in fibroblast cultures, at experimental conditions where the viability of both control and tau-overexpressing fibroblasts was not compromised (Miyoshi et al., 2006; Loo and Halliwell, 2012).

In agreement with the classical role of tau, total tau evidenced with the Tau5 antibody was mainly detected in the cytosol. The signal of total tau increased upon oxidative stress. Such signals were even higher upon tau overexpression with no changes in their localization. In contrast, antibodies directed against phosphorylated tau also showed nuclear localization of Tau; which was accentuated in response to oxidative stress. Moreover, tau overexpression conditions allowed observing more closely dynamic changes on the localization of the AT8 phosphorylated form (AT8). AT8-Tau was mainly located in the cytosol but upon oxidative stress its levels increased in the nucleus. This pattern was also observed upon tau overexpression but at higher magnitude. The anti-AT8 serum showed similar findings with higher background, which is attributed to the low specificity given by the use of unpurified polyclonal antibodies. These findings suggested that the tau protein was phosphorylated and internalized into the nucleus in response to oxidative stress, which has been reported in neurons (Audrey et al., 2011).

Taken together, the findings of the present study suggest that the phosphorylation state and accumulation levels along subcellular compartments of Tau are modified upon oxidative stress by mechanisms that remain to be characterized; which is in accordance with reports in neurons (Audrey et al., 2011). In fact the presence of hyperphosphorylated tau in mouse (Klenyaeva et al., 2014) and human (Corlier et al., 2015) fibroblasts has been described. It is believed that the tau protein plays an important role in the compensatory mechanisms of the cell upon oxidative stress and may mediate epigenetic mechanisms (Mastroeni et al., 2011; Frost et al., 2014; Sanchez-Mut and Gräff, 2015). The fibroblasts model could serve as a system simulating the events occurring at the early stage of taupathies (Klenyaeva et al., 2014), thus this model will generate new insights on the tau protein mechanisms involved in the onset of neurodegenerative disorders.

Current experimental approaches to study AD physiopathology comprise neurons in culture and transgenic animals (Calissano et al., 2009; LaFerla and Green, 2012). Limitations of such systems include difficulties to cultivate neurons *in vitro* and the long time required to induce pathological signals in test animals. In contrast, fibroblasts are extraneuronal, easy to obtain cells that grow in a reasonable time and could serve as a practical model to study AD physiopathology (Choi et al., 2016). Fibroblasts cultures provide some technical advantages such as easy obtention and isolation from skin biopsies, in contrast to brain or nervous tissues where tau pathology is primarily developed. Moreover, fibroblasts share some phenotypic characteristics with neurons in AD and Parkinson (Auburger et al., 2012) and transfection can be efficiently accomplished by a number of methodologies (Bayreuther et al., 1991).

Our study constitutes the first report on the detection of distinct forms of tau in cultured fibroblasts subjected to overexpression and oxidative stress and represents a step forward in the implementation of models to study, at the cellular level, the behavior of this protein of key relevance in the physiopathology of proteinopathies.

## Author contributions

AI established fibroblasts cultures and performed transfection and immunodetection assays. SR and MJ designed the study and wrote the manuscript. MJ supervised data analysis. IR diagnosed the patients and obtained the samples from patients and subjects. BB obtained the tau expression vector. EC collaborated on immunohistochemistry analysis and data analysis. EM performed Western blot analysis. All authors discussed the results, read and approved the final version of the manuscript.

### Conflict of interest statement

The authors declare that the research was conducted in the absence of any commercial or financial relationships that could be construed as a potential conflict of interest. The reviewer EBCG and handling Editor declared their shared affiliation.
