# Supplementary material for: Oxidative Stress Modifies the Levels and Phosphorylation State of Tau Protein in Human Fibroblasts
Source: Front Neurosci. 2017 Sep 7;11:495. doi: 10.3389/fnins.2017.00495 (PMC5594088; doi:10.3389/fnins.2017.00495)
Supplement: Supplementary file 1 [file DataSheet1.DOCX]

**Supplementary figure 1**

**A**


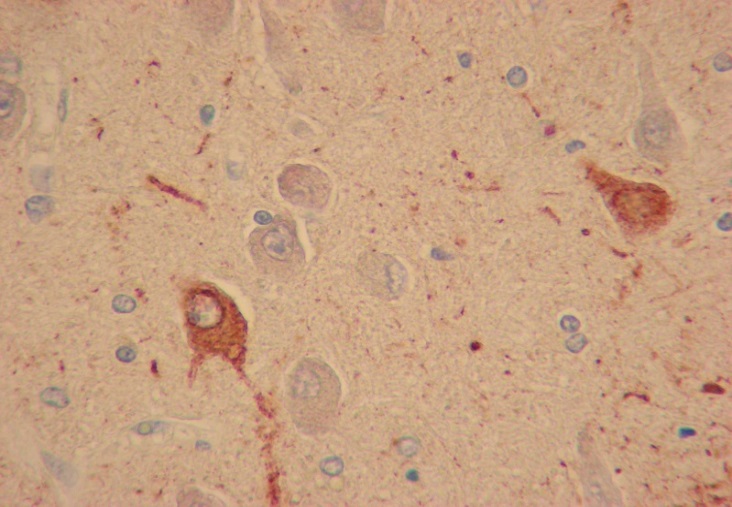


20X

**B**


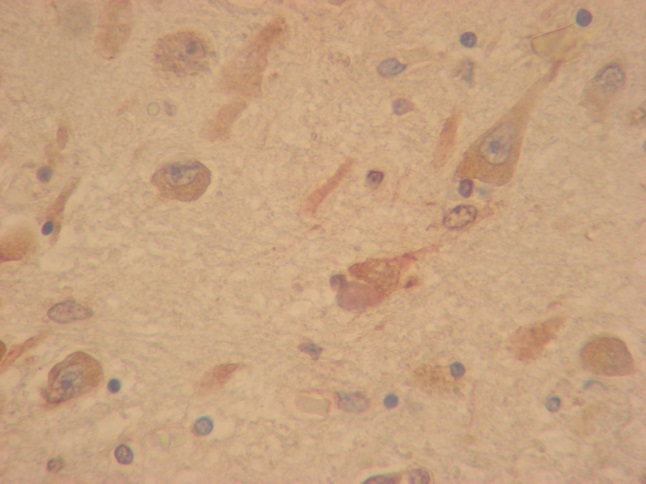


20X

**Figure S1. Assessment of anti-AT8 serum reactivity.** Histological sections from an AD patient´s brain were analyzed by conventional immunohistochemistry techniques labeling with either AT8 commercial antibody (A) or the in house developed anti-AT8 mouse serum (B).
